# Properties of Ambient-Cured Normal and Heavyweight Geopolymer Concrete Exposed to High Temperatures

**DOI:** 10.3390/ma12050740

**Published:** 2019-03-04

**Authors:** Farhad Aslani, Zohaib Asif

**Affiliations:** 1 Senior Lecturer, School of Civil, Environmental, and Mining Engineering, University of Western Australia, Perth, WA 6009, Australia; 2 Adjunct Associate Professor, School of Engineering, Edith Cowan University, Perth, WA 6027, Australia; 3 Master Student, School of Civil, Environmental, and Mining Engineering, University of Western Australia, WA 6009, Australia; 22187053@student.uwa.edu.au

**Keywords:** geopolymer concrete, heavyweight geopolymer concrete, magnetite aggregates, normal-weight coarse aggregates, high temperatures

## Abstract

Ambient-cured heavyweight geopolymer concrete (HWGC) is a new type of concrete that combines the benefits of both heavyweight concrete (HWC) and geopolymer concrete (GC). HWGC provides proper protection from the sources that emit harmful radiations in medical and nuclear industries. Furthermore, HWGC may also be used in offshore structures for pipeline ballasting and similar underwater structures. In this study, heavyweight aggregates (magnetite) have been used and replaced by normal-weight coarse aggregates in GC at volume ratios of 50, 75, and 100% to attain heavyweight classification according to British standards. This study investigates the impacts of high temperatures on standard ambient-cured geopolymer concrete and ambient-cured HWGC through its residual properties regarding compressive and tensile strengths, mass loss, spalling intensity, and flexural strength. The residual properties were examined by heating 100 × 200 mm cylinder specimens to 100, 300, 600, and 900 °C. The results indicated that the maximum compressive strengths of 40.1 and 39.0 MPa were achieved by HWGC at 300 and 100 °C, respectively. The overall result shows that the strength of HWGC increases by increasing magnetite aggregate proportion, while the mass loss, intensity of spalling, and loss of strengths is proportional to temperature after a certain point. Minor spalling with holes and cracking was observed only at 900 °C in HWGC.

## 1. Introduction

Portland cement has been around for almost 200 years; however, the Romans and Egyptians were using cement-like materials centuries before Joseph Aspdin would patent Portland cement [1]. Nowadays, ordinary Portland cement (OPC) is the second most consumed product on Earth, second only to water, and is expected to increase from 2.55 billion tonnes in 2006 to 3.7–4.4 billion tonnes by 2050 [2]. This gives rise to an increasing need to find an environmentally friendly alternative to Portland cement in order to reduce carbon dioxide emissions and promote green concrete technology by utilizing various by-product materials such as fly ash and blast furnace slag. However, many studies have indicated the potential benefits of fly-ash-based geopolymer concrete over the OPC concrete in the last few years [3]. Hence, geopolymer concrete has shown the potential to replace OPC by reducing the amount of carbon emissions up to 80%, while still maintaining high strengths comparable to that of OPC [4].

Geopolymer concrete, which is also known as alkali-activated or inorganic concrete, is a different kind of concrete, using different chemistry to that found in OPC concrete. The historical backdrop of geopolymer started with the first patented by German chemist and engineer Kűhl in 1908, where a combination of alumina and silica content (vitreous slag) with an alkali (alkali sulfate or carbonate) source led to the development of solid material comparable to OPC [5]. The improvement of this binder was enhanced by Purdon where he published an important journal with respect to the achievement rates of strength and the final strength that is equivalent to the OPC activated by solution of sodium hydroxide and calcium hydroxide combined with different sodium sources tested under different types of blast furnace slags [6,7]. Purdon faced a problem with the handling of a high concentrated solution and the sensitivity of total water added from the activation conditions [8]. Research on alkali-activated binders then broadened eastward for a couple of decades, including the Soviet Union and China, where a new alternative material as a substitution of OPC was needed [9]. Glukhovsky [10] from the Soviet Union presented a theoretical basis and development of alkaline cements that leads to the use of material called alkaline cement. Alkaline activation products in Western countries saw no advancement until the last development by Purdon in 1979 [11]. Joseph Davidovits referred to an aluminosilicate-based material as a geopolymer, and he defined it as a three-dimensional, short range order inorganic polymer that forms when high concentrated aqueous alkali hydroxide-silicate solution is added to the alumino-silicate materials [12]. By-product materials such as fly ash and slag are main sources of alumino-silicate materials. The properties of alkali-activated by-product rely on many factors such as the chemical composition of the binders, the type of alkali activators, the concentration of the activator, the curing condition, and the water content [13].

According to British standards [14], concrete is termed heavyweight when the density of the sample reaches an oven dry density of 2600 kg/m^3^, as opposed to normal weight concrete, which has a density of 2400 kg/m^3^. The typical aggregates used to achieve heavyweight concrete are magnetite, barite, hematite, limonite, and limenite [15]. Due to the addition of such aggregates, concrete can achieve densities of up to 8,900 kg/m^3^ depending on the type of material [16]. However, magnetite seems the most practical since it is found abundantly in Western Australia alongside hematite, and, due to the higher iron content found within hematite, magnetite seems to be better suited in the use of heavyweight concrete [17]. Heavyweight concrete has applications in the medical and nuclear industries, where radiation shielding is required. Furthermore, offshore applications have used heavyweight concrete to provide stability to pipelines and other underwater structures. Horszczaruk et al. [15] found that cement mortars containing magnetite offered the highest thermal stability, further justifying the use of magnetite over other heavyweight aggregates in this study. Additionally, Horszczaruk et al. [18] found that the use of magnetite in concrete resulted in a lower rate of deterioration of up to two times that of normal-weight coarse aggregate samples after exposure to high temperatures. The inclusion of heavyweight aggregates in concrete shows a reduction in the thickness required for sufficient radiation shielding of up to 40% compared to concrete with a normal aggregate [18]. However, the tendency of aggregate to segregate in a concrete mixture increases with the increase in density of the aggregates. Therefore, chemical admixtures can be used to control the rheological behavior of heavyweight concrete to control segregation and bleeding.

Currently, the most common binder used in the formation of geopolymer is fly ash. Fly ash geopolymer has been shown to have better mechanical properties and durability compared to OPC [3,19]. In the geopolymerization process, the amount of calcium content in fly ash was found to possess a significant impact on the resulting hardened geopolymer [20]. Oh et al. [21] and Winnefeld et al. [22] stated that class-C fly ash showed lower properties in terms of strength development and binder phase evolution compared to class-F fly ash. Meanwhile, some researchers have affirmed that class-F fly ash as a source material is preferable to class-C fly ash. However, Deventer et al. [23] proved that the presence of higher CaO in class-C fly ash causes a disruption in the geopolymerization process and has the potential to change the existing microstructure of the geopolymer, further justifying the use of class-F fly ash over class-C fly ash in this study. Moreover, class-F fly ash has better resistance against sulfate attack and lowers the heat hydration and heat generation of concrete’s rate than class-C fly ash [24].

Notable studies have been done on the fly ash geopolymer combined with some additional materials [25]. The suitability of fly-ash-based geopolymer blended with ground granulated blast furnace slag has been reported by Dombrowski et al. [26]. The inclusion of GGBFS into fly-ash-based geopolymers resulted in quicker setting times and higher strength, resulting in creating a concrete suitable for ambient curing conditions [27]. Wardhono et al. [28] found out that the mixes containing larger portions of ground granulated blast furnace slag ratios of up to 50% produced the best strength results under ambient curing, albeit providing a much lower setting time. Nath et al. [20] investigated the properties of fly-ash-based geopolymer concrete with different proportions of additives such as GGBFS, OPC, and calcium hydroxide (CH). The maximum compressive strength was achieved with the 10% addition of GGBFS in fly-ash-based geopolymer after 28 days at ambient curing conditions. However, Somna et al. [29] found out that the fly-ash-based geopolymer can be improved by increasing the reactivity of fly ash; i.e., by increasing fineness. Moreover, the addition of calcium oxide and calcium hydroxide as a replacement of fly ash improves the mechanical properties at ambient curing [30].

Alkali activator also significantly influences the geopolymerization process. Different alkali activators have been used in geopolymer concrete, such as alkali hydroxides, alkali silicates, alkali carbonates, and alkali sulfate [5]. Currently, alkali silicate is an important chemical compound that has been used as a good activator to a binder in geopolymer concrete. Na silicates are the most often used activators because of their low cost compared to K-silicate solutions. Alkali concentration has been shown to be one of the main parameters in the contribution to the performance of geopolymer concrete [5]. Many researchers have developed geopolymer concrete by combining an activator between alkali silicates and alkali hydroxides with appropriate ratios [31,32]. Furthermore, the ratio between alkali silicate and alkali hydroxide plays an important role in the mechanical properties of geopolymer concrete. Palomo et al. [33] found that a compressive strength in the range 35–40 MPa will be produced from a reaction of different fly ash types with an alkali activator (NaOH) in the range 8–12 mol/L cured at 85 °C in 24 h, while the compressive strength (with the same conditions) will increase up to 90 MPa when alkali silicate (Na_2_SiO_3_) is added with a SiO_2_/Na_2_O ratio of 1.23. Similarly, Pradip et al. found significant compressive strength by using alkaline solution prepared where the Na_2_SiO_3_/NaOH ratio = 2.5 [20], further justifying the use of NaOH with sodium silicate as an alkaline reactor in this study.

The polymerization reaction is highly influenced by curing conditions. Curing temperature impacts the microstructural and mechanical development of geopolymer concrete. Normally, the polymerization reactions in low calcium fly ash based geopolymer concrete are accelerated at high temperatures which enhances mechanical and durability properties of concrete, compared with geopolymerisation reactions at ambient curing conditions [34]. However, the polymerization process and resulting products may also be affected by other factors such as the type and properties of alumino silicate sources and the composition of the alkaline solution [9]. Class-F fly-ash-based geopolymers outperform the conventional concrete in fire, due to their ceramic-like properties [35]. The mechanical strength of geopolymer changes due to high-temperature-induced structural and phase composition changes in the material [36]. Structural changes include sintering, densification, melting, cracking, and pore size changes. Phase composition changes include crystal growth, crystal destruction, hydration, and geopolymer paste decomposition and release of free Si, Al, and alkali. However, thermal dilation of secondary phases such as crystalline impurities and aggregate during exposure also affects the mechanical strength [37]. Moreover, the improvement in the interconnectivity through sintering could increase mechanical strength, which, in some cases, can be more than five times the strength at ambient temperature [38]. According to Rickard et al. [39], sintering can be defined as the “heal” of the crack induced during the dehydration phase (the phase in which water escaping caused the structural damage) and hence improve the mechanical properties of the materials.

The strength development of geopolymer concrete is heavily influenced by the water content. It is crucial to control the amount of moisture going into a mix [27,40]. A water-to-binder ratio in geopolymer is best kept around 0.2 to achieve the minimum needed for workability [27,41]. Compressive strengths were shown to decrease exponentially when the water-to-solid ratio increased from around 0.15 to 0.5 [42]. Admixtures can be used to improve and manipulate the fresh properties of a concrete mix to better suit the concrete for a specific use [43]. The use of superplasticizers and water reducing admixtures can cause a reduction in the amount of water in a mixture by up to 30 and 12%, respectively [44]. Both types of admixtures can either show an increase or decrease in setting time depending on how it reacts to the mix with the water reducing admixture, causing an initial setting time to increase by 3 h, and the superplasticizer can either decrease or increase setting time by up to 1 h. The addition of superplasticizers can show an early concrete strength increase by 50–75% and an increase in workability on the fresh properties of concrete [44].

It should be noted that numerous studies have been conducted on the thermal characteristics of standard geopolymer concrete [27], but no study has examined the effects of prolonged exposure to high temperatures on the physical and mechanical properties of heavyweight geopolymer concrete in comparison with standard geopolymer concrete. The research presented here will explore the ability of materials to produce standard GC and HWGC, together with binders, fillers, sand, SP, and others, using locally available materials in Australia. This research will help in standardizing local materials and obtaining an idea of the strength it can generate and its durability when exposed to fire at high temperatures. Therefore, it will promote the sustainable use of GC and HWGC in Australia for radiation shielding purposes and other offshore structures.

## 2. Experimental Study

### 2.1. Materials

#### 2.1.1. Fly Ash

The primary binder material in geopolymer concrete is fly ash, which provides strength and improves the workability of concrete due to their spherical glassy shape particles [45]. Low-calcium fly ash of class-F in accordance with the requirements of ASTM-C618 [46] was used as the primary binder material. The chemical and physical compositions of fly ash are given in Table 1.

#### 2.1.2. Ground Granulated Blast Furnace Slag

The inclusion of GGBFS in fly-ash-based geopolymer concrete resulted in quicker setting times and higher strength, resulting in creating a concrete suitable for ambient curing conditions [27]. Mixes containing larger portions of ground granulated blast furnace slag ratios of up to 50% produce the best strength results under ambient curing [28]. GGBFS complies with AS-3582.2 [47]. The properties of GGBFS are given in Table 2.

#### 2.1.3. Alkaline Solution

Alkaline solution was prepared by adding sodium hydroxide (NaOH) and sodium silicate (Na_2_SiO_3_) solutions. Sodium hydroxide liquid was prepared in the laboratory by mixing 98–99% pure sodium hydroxide pellets, collected from a local producer and with a density of 2.13 g/cm^3^, with normal tap water. The N-grade sodium silicate solution used in this study, collected from a local producer, had a molecular ratio of SiO_2_ to Na_2_O of 3.2 with a 1.39 g/cc density (SiO_2_ = 28.6%, Na_2_O = 8.9% and H_2_O = 62.5% by weights). Alkaline solution was prepared by mixing sodium hydroxide and sodium silicate solutions together and left at room temperature to cool down for 1 h prior to mixing.

#### 2.1.4. Natural Aggregates

**Figure 1:**
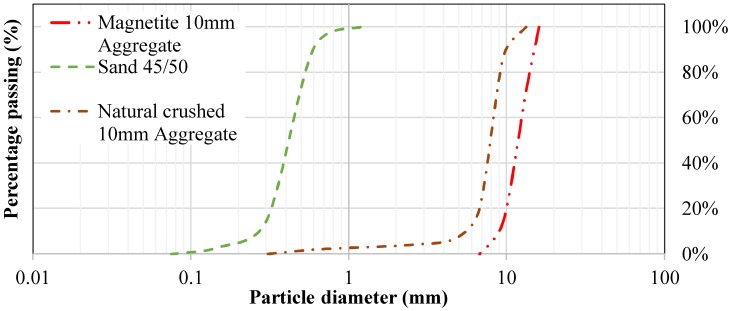
Grading curve of sand, 10 mm natural aggregate and 10 mm magnetite aggregate.

In this study, 10 mm naturally crushed aggregates were used as normal-weight coarse aggregates. Fine AFS 45-50 silica sand obtained from Rocla Quarry Products, Redcliffe, Western Australia was used in this study. The sampling methods and testing of these aggregates were performed according to AS-1141 [48]. Results are shown in Table 3, Table 4 and Table 5 and the particle distribution curve is shown in Figure 1.

#### 2.1.5. Heavyweight Aggregates

Heavyweight aggregate that was used in heavyweight geopolymer concrete mixes consisted of magnetite. Magnetite is a low grade and unrefined iron ore with a density of approximately 1.4 times that of regular aggregates. Magnetite was chosen due to its availability in Western Australia, as well as its ability to maintain high compressive and tensile strengths. Particle sizes from 6–10 mm were used in this research, and their distribution is shown in Table 6a. However, the chemical and physical properties of magnetite aggregate can be found in Table 6b. It is stated in previous studies that the presence of iron in geopolymers contributes in the formation of Fe–O bonds within the geopolymer matrices; replacing Al^3+^ by Fe^3+^ in octahedral sites causes an increase in strength by enhancing the geopolymer gel matrix [49,50,51]. Therefore, the inclusion of magnetite aggregate can cause an increase in the strength of geopolymer concrete. However, the particle size distribution of magnetite aggregate can be seen in Figure 1.

#### 2.1.6. Chemical Admixtures

In this experimental study, superplasticizer admixture (SP) was used that satisfies Type SN chemical admixture according to AS-1478.1 [52]. It is designed to improve the flow properties of concrete by lowering the viscosity and yield stress of fresh concrete. The significant improvement in the rheological behavior of the geopolymer concrete was observed with the addition of SP in this experimental study. 

### 2.2. Mix Proportions

In this experimental study, to investigate the performance of a standard geopolymer concrete mix, M1 was prepared by using normal-weight coarse aggregates (10 mm naturally crushed aggregates), and three HWGC mixtures M2, M3, and M4, were prepared replacing 10 mm normal-weight coarse aggregates with 10 mm magnetite aggregates at 50, 75, and 100% by volume respectively. In total, four mixes were designed as shown in Table 7. The mix design set was based on the control mix (M1), which included 400 kg/m^3^ binder content with naturally crushed aggregates and a water-to-binder ratio of 0.123, which is less than that suggested to achieve the minimum needed workability, but the sodium hydroxide solution is made by combining pure solid sodium hydroxide pellets with water, and this gives a water content of 0.2 times of total dry binder within the whole mix to make up the minimum needed to achieve workability. The binder composition of the mixes was composed of 90% fly ash and 10% GGBFS. Alkaline solution was used as 40% of the total binder, and the ratio of Na_2_SiO_3_/NaOH was 2.5. The concentration of sodium hydroxide was 14 mol/L in all mixtures. All concrete mixtures were conducted with constant binder proportions, alkaline solution, water content, and admixture. The same mix design set was used for mixes M2, M3, and M4 containing the replacement of normal-weight coarse aggregates with magnetite aggregates at 50, 75, and 100% by volume, respectively.

### 2.3. Casting and Curing of Samples

For mixing, all saturated surface dry aggregates, sand, and solid binder materials (fly ash and GGBFS) were collected in a pan mixer and then dry mixed for up to 5 min. Once the solid materials were mixed comprehensively, alkaline solution was then added followed by water and superplasticizer and allowed to mix for another 5 min. Cylindrical moulds of 100 × 200 mm were cast for compressive and tensile strength and stress–strain tests. Rectangular prism moulds of 450 × 100 × 100 mm were cast to determine flexural strengths. Moulds were filled and compacted using a combination of rodding, vibrating, and tapping with a hammer to ensure any voids within the mould were filled. The moulded samples were cured in room conditions (20 ± 2 °C). Once hardened, the specimens were de-moulded after 24 h of casting. After removing from moulds, the samples were left in a humidifying room at a temperature of 20 ± 2 °C until tested after 7 and 28 days. This process was conducted for the standard geopolymer control mix M1 and repeated for M2, M3, and M4 where normal-weight coarse aggregates were replaced with magnetite aggregates at 50, 75, and 100% by volume.

### 2.4. Test Methods

#### 2.4.1. Mechanical Properties

##### Compression Test

Once the moulds reached their designated curing times, three representative concrete cylindrical samples from each batch were chosen and tested at every temperature. The hardened properties were measured by heating the designated samples at 5 °C/min to temperatures of 100, 300, 600, and 900 °C. The specimens remained in furnace for 1 h and then allowed to cool at room temperature for 24 h before the residual strength tests were conducted. Three 100 × 200 mm cylindrical samples were placed underneath a Baldwin compression/tension machine, which applied load at a rate of 0.2 kN/s to the sample overtime until failure at each age of 7 and 28 days. The results were recorded and a maximum load at failure used to determine the compressive strength of the specimen. The testing procedure follows AS-1012.14 [53]. The three tested samples were weighed, and their dimensions measured to obtain their hardened density in accordance with AS-3582.3 [54]. This process was conducted for the mix M1 and repeated in the same manner for all other mixes.

##### Tensile Test

Splitting tensile tests were conducted on three 100 × 200 mm cylindrical specimens using the Baldwin compression/tensile machine at 20, 100, 300, 600, and 900 °C temperatures on testing ages of 7 and 28 days. Samples were placed between two steel square section bars and compressed at a rate of 0.2 kN/s until failure. The tests were conducted in accordance with AS-1012.10 [55]. Results were recorded and maximum load at failure used to determine the tensile strength of the specimen. This process was repeated for every mix sample.

##### Flexural Test

The flexural strength test was conducted in accordance with AS-1012.11 [56]. Three rectangular prism specimens of size 450 × 100 × 100 mm were tested under four-point loading to measure the flexural strength. The residual flexural strengths were examined for specimens at 20 °C on their specified curing ages. The maximum load at failure was used to calculate the flexural strength of the sample. This testing process was used for all mix samples.

##### Compressive Stress–Strain Test

100 × 200 mm cylindrical samples were tested for their compressive stress–strain curve at 28 days. Specimens were attached with 60 mm horizontal strain gauges to examine the stress–strain curves as per AS-1012.17 [57].

#### 2.4.2. Properties of Fresh Concrete

A slump test was conducted on the control mix and the heavyweight geopolymer samples to show the effect of the heavyweight aggregate on the flow of the concrete. The slump flow test was conducted using an Abrams cone in accordance with AS-1012.3.5 [58]. 

#### 2.4.3. Spalling and Mass Loss

Once moulds reached their designated curing ages, they were removed from the curing room and weighed before being placed into a furnace for heating. Specimens were then heated at 5 °C/min to 100, 300, 600, and 900 °C, similar to [59,60], and removed from the furnace. The samples were then weighed again and observed for any spalling that may have occurred during the heating process. The measurement of the mass of samples before and after heating was done in accordance with AS-1012.12.1 [61]. The recorded weights were then used to determine overall mass loss at each temperature. This process was conducted for the control mix M1 and repeated for M2, M3, and M4. Spalling of the specimens was physically observed for the formation of cracks, loss of parts, and holes. The effects of spalling vary depending on the purpose for which concrete is used.

## 3. Experimental Results

### 3.1. Properties of Fresh Concrete

**Figure 2:**
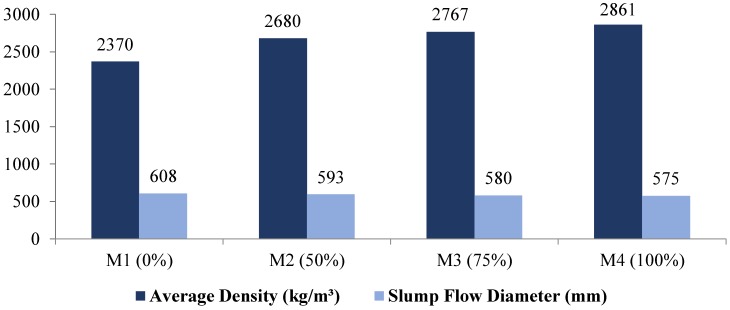
Fresh property test results.

**Figure 3:**
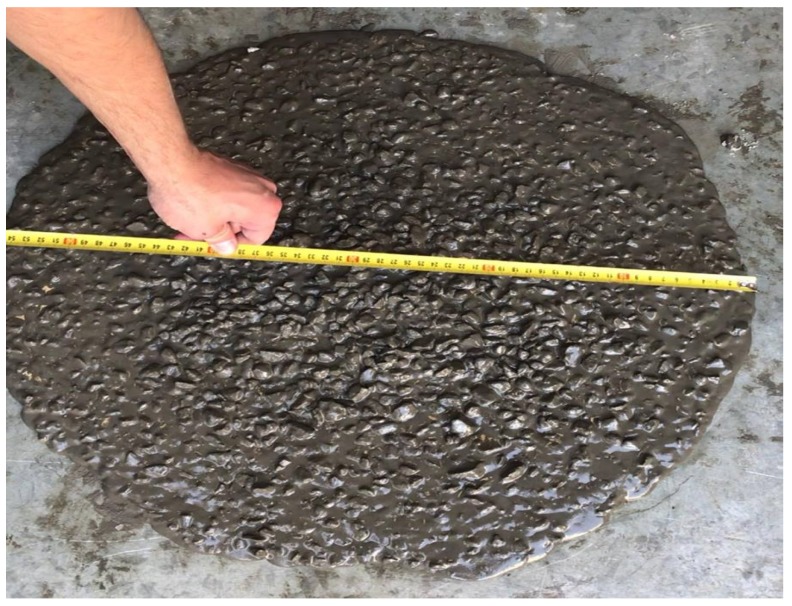
Fresh property slump flow test.

The results of fresh property testing, including slump flow diameter of all mixes with respective dry densities following the hardening of samples are presented in Table 8 and Figure 2 and Figure 3.

The constant amount of superplasticizer was used in all mixes to examine the characteristics of slump flow diameter of geopolymer mixes with increasing densities. The superplasticizer increases the flow of mixture by lowering the viscosity. According to ASTM-C143/M-03 [62] and Gosh & Gosh [63], in their table of geopolymer concrete workability criteria, the slump flow for high workability geopolymer concrete is >250 mm. All mixes satisfied the geopolymer criteria for flowability. However, these mixes showed a decrease in slump flow diameter at increasing magnetite replacements. It can be seen in Figure 2 that, as the percentage of magnetite aggregates increases, the slump flow diameter decreases. Hence, this decrease can be attributed to the difference in the water absorption rate in normal-weight coarse aggregates and that in magnetite aggregates, where the latter absorbs more water than normal-weight coarse aggregates. For the M1 mix, slump flow was recorded as 607.5 mm and with a replacement of 50, 75, and 100% magnetite aggregate. Decreases in slump flow of 2.5, 4.5, and 5.4% were found in the M2, M3, and M4 mixes respectively. Furthermore, the average dry density of M1 reached about 2370 kg/m^3^. With 50% magnetite replacement (M2), density increased to approximately 2680 kg/m^3^, which indicates that heavyweight concrete status has been achieved. Further replacement in M3 and M4 mixtures displayed a dry density of 2765 kg/m^3^ and 2860 kg/m^3^, respectively. 

### 3.2. Hardened Properties

**Figure 4:**
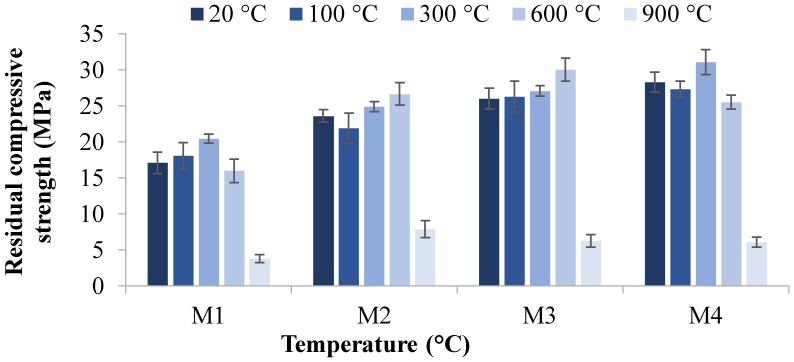
Residual compressive strength versus temperature after seven days.

**Figure 5:**
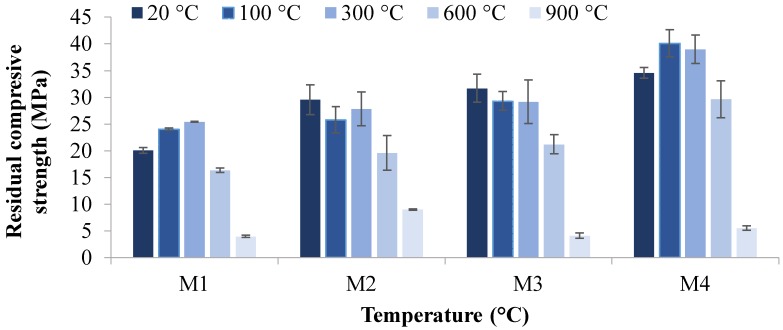
Residual compressive strength versus temperature after 28 days.

**Figure 6:**
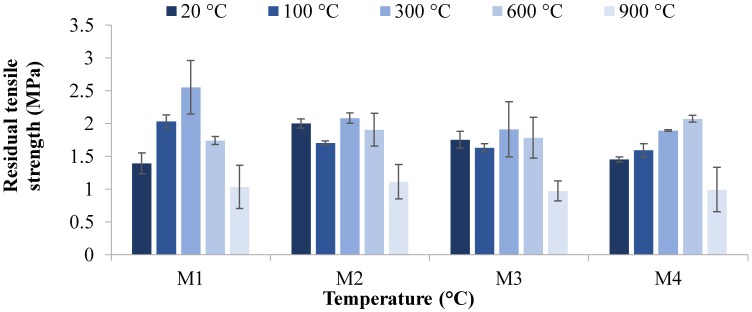
Residual tensile strength versus temperature after seven days.

**Figure 7:**
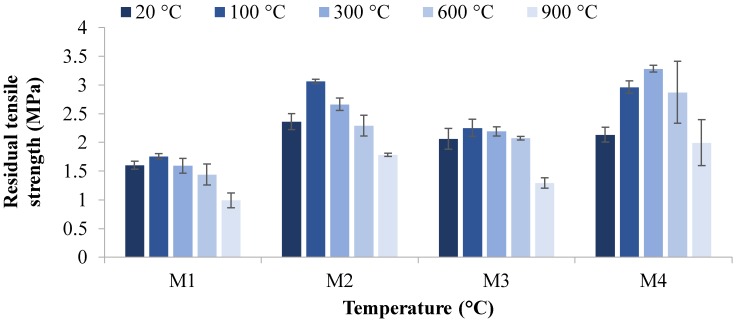
Residual tensile strength versus temperature after 28 days.

**Figure 8:**
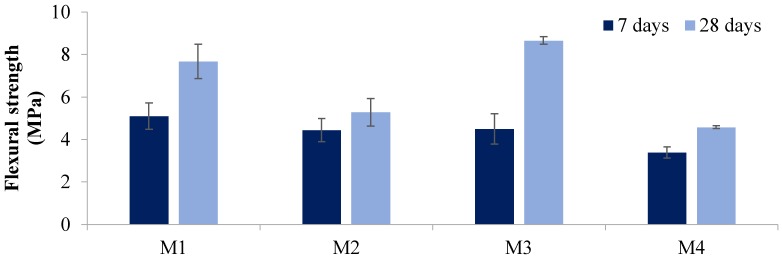
Flexural strength versus days at 20 °C.

The residual compressive strength and residual tensile strength at 20, 100, 300, 600, and 900 °C temperatures are given in Figure 4, Figure 5, Figure 6 and Figure 7. The residual flexural strength at 20 °C is shown in Figure 8.

#### 3.2.1. Compressive Strength

Figure 4 and Figure 5 show the residual compressive strength of M1, M2, M3, and M4 mixes at high temperatures. The maximum compressive strengths of 40.1 and 39.0 MPa were obtained for M4 at 100 and 300 °C, respectively. It has already been reported that the geopolymer concrete gives higher strength when cured at higher temperatures. Thus, densification of geopolymer appears to occur at 100 and 300 °C, resulting in an increase in compressive strength. Particles containing hydroxyl ions (OH^−^) can be bound to each other by a dehydration reaction, releasing water to form a larger particle at 100 and 300 °C temperatures. Fly-ash-based geopolymer has a chemically bonded hydroxyl group to silicon (Si–OH), which is released at higher temperatures and gives a Si–O–(Si or Al) structure, increasing the connectivity, strength, and stability [64,65,66]. However, severe macro-cracks were observed for samples when the temperature was increased from 600 to 900 °C. This is attributed to the destruction of the cellular structure of geopolymer due to dehydration damage, dimensional instability, and sintering [39,67]. It is also evident in Figure 4 and Figure 5 that, as the percentage of magnetite aggregates increases, the compressive residual strength also increases. This indicates fewer voids and less porosity in the mixture due to replacements with magnetite aggregate, resulting in high compressive strengths. It should be noted that there is a small increase in strength when the age of specimens increased from 7 to 28 days, which shows the rapid chemical reactions in the geopolymerization process, which enables the geopolymer to gain their extensive strengths in early ages. [12,68].

In seven-day sample testing, as shown in Figure 4, all specimens showed an increase in compressive strength after being exposed to 100 and 300 °C, which is attributed to the densification process at low elevated temperatures where the Si–O–(Si or Al) structures develop [64]. With the increase in temperature to 600 °C, M2 and M4 specimens showed an increase in compressive strength. This suggests that the sintering of unreacted material increased the mechanical strength due to stronger bonding between the particles [67]. However, the loss in strength at 900 °C is consistent with the photographs in Figure 13, as these samples exhibited extensive macrocracking. 

Similarly, as shown in Figure 5, after being exposed to a temperature of 100 and 300 °C, all tested specimens exhibited an increase in compressive strength in the 28-day sample testing. Interestingly, samples at 600 °C exhibited enormous micro cracks on the surface but still retained significant compressive strengths. The sintering process may be considered the main reason for keeping strength even with microcracking on the surface of samples at 600 °C. Moreover, it was found that the compressive strengths of all mixes were severely reduced when samples were exposed to 900 °C, developing macro-cracks due to the destruction of the cellular structure of the geopolymer [39,67].

The results revealed that, in all mixes, the strength increases with the improvement in interconnectivity through a densification process at 100–300 °C. However, when the temperature increased from 600 to 900 °C, severe macro-cracks developed due to the cellular destruction of geopolymer chemistry, dehydration damage, dimensional instability, and sintering, causing reduction in strengths.

#### 3.2.2. Tensile Strength

Figure 6 and Figure 7 show the residual tensile strength of M1, M2, M3, and M4 mixes at high temperatures. The maximum tensile strengths of 3.3 and 3.1 MPa were obtained for M4 at 300 °C and M2 at 100 °C, respectively, after 28 days. 

The trend of tensile strength (Figure 6 and Figure 7) is similar to that of compressive strength (Figure 4 and Figure 5). It is evident in Figure 6 and Figure 7 that the majority of specimens showed an increase in tensile strength after being exposed to 100 and 300 °C. With the increase in temperature to 600 °C, all specimens showed a decrease in tensile strength. Moreover, a severe decrease in tensile splitting strength was observed when the specimen was exposed to 900 °C.

With respect to the increase of magnetite aggregate proportions in mixes, a different trend was observed. An increase in strength can be seen in Figure 6 and Figure 7 as magnetite aggregate was increased from 0 to 50%, sowing fewer voids and less porosity and causing an increase in strength. This was followed by a decrease in tensile splitting strength when the magnetite replacement increased from 75 to 100%. The tensile splitting strength of M2 was 30.5% higher than the M1 mix with the replacement of 50% magnetite aggregates under ambient temperature after seven days. However, the tensile strength was 12.5% and 27.5% lower than that of the M2 mix with the 75% and 100% increases in magnetite aggregates, respectively, under ambient curing conditions at seven days.

The results revealed that, among all mixes, the highest tensile strength was achieved in M4 at 300 °C and M2 at 100 °C. The highest strengths at high temperatures are due to the improvement in interconnectivity through the densification process, enhancing the geopolymer grains. However, with the rise in temperature to 600 °C, the strength of all mixes was slightly affected, followed by an intense decline at 900 °C. This decrease in strength is mainly due to the destruction of the cellular structure of geopolymer started after 600 °C, causing severe cracks on the surface, which leads to an immense reduction in strength. Similarly, the tensile strength was increased, as magnetite replacement reached 50%. With the increase of the 75–100% replacement of magnetite aggregates, a decrease in tensile strength was observed. 

#### 3.2.3. Flexural Strength

Figure 8 shows the effect of aggregate replacement on the flexural strength of the geopolymer samples at 20 °C. There was no clear trend occurring in this set of results. The 0% aggregate replacement after 28 days showed a drop in flexural strength with 50% magnetite aggregate replacement. This then increased with 75% (M3) magnetite replacement and dropped again after 100% (M4) magnetite aggregate replacement was achieved. The lowest flexural strength after 28 days occurred with 100% magnetite aggregate replacement and only reached about 4.5 MPa. However, the highest flexural strength of 8.6 MPa was achieved by the M3 samples.

#### 3.2.4. Compressive Stress–Strain Behavior

**Figure 9:**
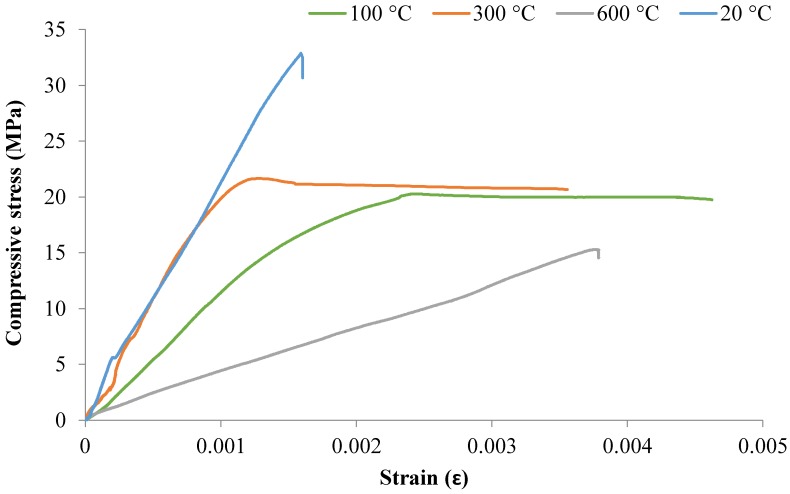
Compressive stress–strain curves for M2 after 28 days at high temperatures.

**Figure 10:**
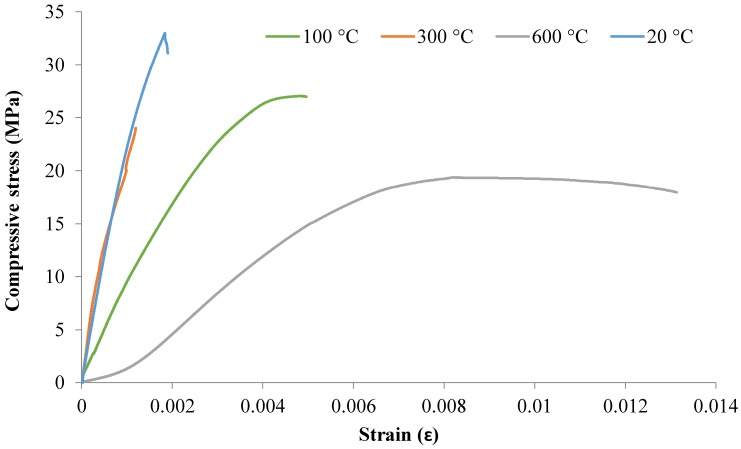
Compressive stress–strain curves for M3 after 28-days at high temperatures.

**Figure 11:**
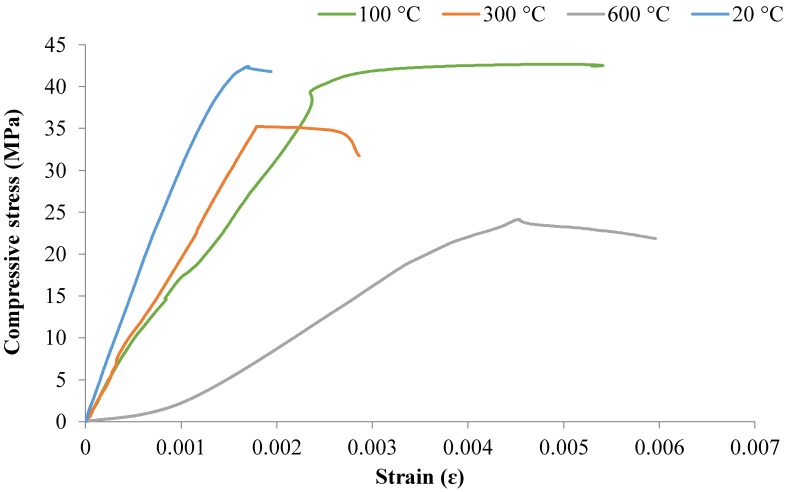
Compressive stress–strain curves for M4 after 28 days at high temperatures.

The compressive stress–strain curves for the mixes M2, M3, and M4 at 20, 100, 300, and 600 °C for 28 days are shown in Figure 9, Figure 10 and Figure 11. The stress–strain curves at 900 °C were not examined because the strain gauges could not stick properly to the samples due to their softness and cracks.

Figure 9, Figure 10 and Figure 11 show that the M2, M3, and M4 mixes exposed to high temperature lost their structural integrity when decreasing their compressive strengths and stiffness, which led to easy deformation. Generally, because of the decrease in compressive strength and the increase in the strain of the concrete, the slope of the stress–strain curve decreased with increasing temperature. The strength of concrete had a significant influence on the stress–strain behavior as temperatures elevated. The cylindrical specimens underwent more spalling when subjected to high temperatures and caused a loss in bond due to a weakening of the material, which led to an increase in strain. It can be seen in the figures that the compressive strength decreased and that peak strain increased with increasing temperatures due to the softening of concrete that occurs.

It should also be noted, in light of Figure 11, that exposure of the M4 mix to 100 °C led to higher compressive strength with higher strain. This provides evidence that exposure to 100 °C may improve the mechanical properties of M4 mixes. The lowest stress of 24 MPa was recorded for the M4 sample with a strain of just over 0.00454. However, the maximum stress of around 42.6 MPa was noted at 100 °C with a strain of over 5406 microstrain.

### 3.3. Mass Loss

**Figure 12:**
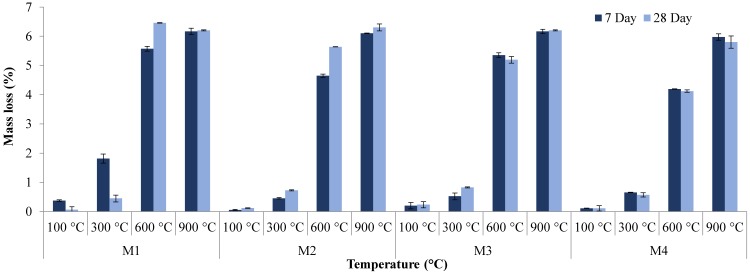
Mass loss versus temperature.

Mass loss was recorded for each sample at 7 and 28 days. Each sample was weighed before and after exposure to high temperatures on their specific ages, and average mass loss was used to determine the percentage of mass loss overall [69,70,71,72,73,74]. Figure 12 and Table 9 show the percentage of mass loss for 7-day and 28-day samples after exposure to temperatures ranging from 100 to 900 °C. Figure 12 shows an increase in mass loss for all types of samples with increasing temperature. However, with the increase in density of samples, a small decrease in mass loss was observed. As the magnetite replacement was 100% in the M4 mix, these samples showed a lower mass loss percentage as compared to M3.

For seven-day samples at 100 °C, the lowest percentage of mass loss was recorded, while at 900 °C the highest was observed. The mass loss of samples at 100 °C averaged less than 0.2%, with a maximum mass loss of 0.38% recorded for the M1 mix. At 300 °C, the average percentage of mass loss increased to 0.86%, but the M1 mix results showed an unusually high percentage compared to other samples with different replacements. The maximum recorded mass loss at 300 °C reached about 1.8%. At 600 °C, a significant increase in mass loss occurred compared to previous temperatures. Average mass loss at 600 °C was 4.94% with a maximum of 5.57% occurring in the M1 sample again. At 900 °C, mass loss percentage was the highest with an average of 6.1% with little deviation in each replacement. The maximum was again recorded for the M1 mix, but this was shared with the 75% replacement at 6.16%. Therefore, for all types of mixes, the mass loss was increased with the increase in temperature.

For 28-day samples, overall the same trend of increase in mass loss was observed with the increase in temperature. However, only in M1 samples was the mass loss at 900 °C less than the sample at 600 °C, which is mainly due to error in measurements. However, at 100 °C, average mass loss totaled 0.135% across all replacements with the highest mass loss occurring for M3, which reached 0.24%. The mass loss increased to an average of 0.64% for samples subject to 300 °C and a maximum mass loss of 0.82% was calculated, which again came from the M3 samples. A huge jump in mass loss occurred when samples were heated to 600 °C with an average of 5.35% being observed. M1 mix samples possessed the highest mass loss at 600 °C with a 6.46% mass loss being recorded. Compared to the 7-day results, mass loss at 100–300 °C was fairly similar to the 24-day results, the 7-day results showing higher averaging, but at 600 °C 28-day samples averaged higher than the 7-day samples. Mass loss was, on average, about 0.4% higher in 28-day samples than in 7-day samples with a maximum mass loss being 0.9% higher than the highest mass loss of the 7-day concrete.

### 3.4. Spalling 

Table 10 reports the effect of temperature on the physical form of the concrete, whether spalling occurred, and any noticeable changes for samples subjected to high temperatures [70,71].

**Figure 13:**
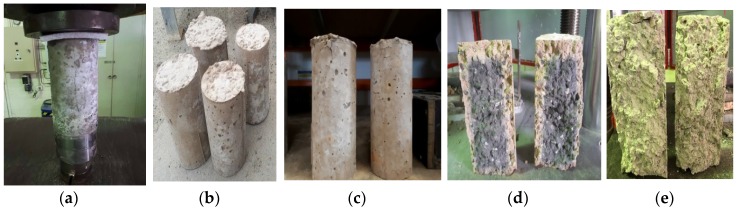
The effect of high temperatures on mix samples at (**a**,**b**) 600 °C and (**c**–**e**) 900 °C.

Temperatures between 100 and 600 °C had little effect on the physical nature of all mix samples. However, a significant change in appearance occurred when all mix samples were exposed to 900 °C, samples began to resemble pottery or ceramic material, and cracks formed towards the top of the samples. The geopolymer samples at 20 °C have a dark grayish color, and as temperature increases it begins to form a lighter gray color at 600 °C, but at 900 °C the color changes to an orange. The effects of temperature at 600 °C are shown in Figure 13a,b, while the effects of temperature at 900 °C on samples can be seen in Figure 13c–e.

### 3.5. Failure Modes

**Figure 14:**
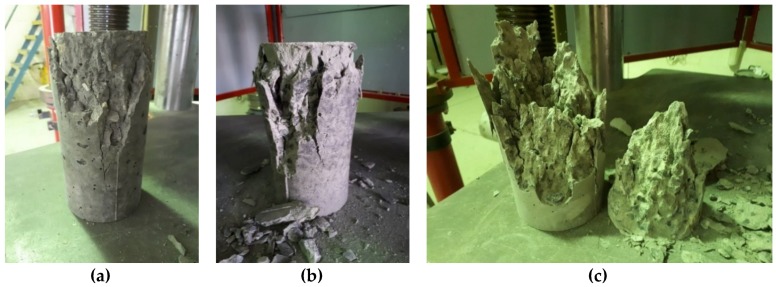
(**a**–**c**) Conical failures of samples subjected to compression testing.

**Figure 15:**
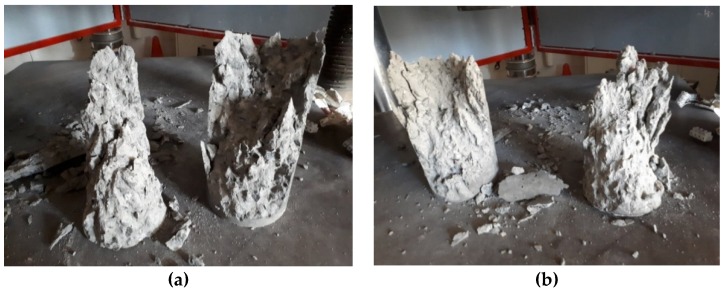
(**a**,**b**) Partial cone/shear failure of samples subjected to compression testing.

**Figure 16:**
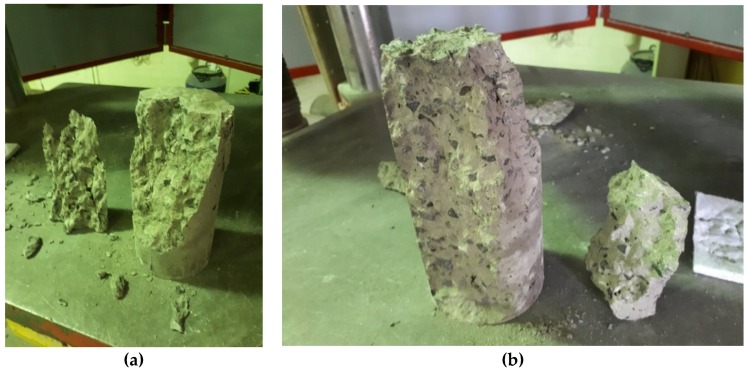
(**a**,**b**) Vertical shear failure of samples subjected to compression testing.

Modes of failure through the duration of this research varied between a conical failure to a partial cone/shear failure and a vertical shear failure. Of the three modes, the conical failure is most preferred, and this was the type of failure for the majority of tests done. However, other modes would suggest a premature failure of a sample, so some results may be lower than expected. Figure 14, Figure 15 and Figure 16 show the three main types of failure reported through the duration of this research project.

**Figure 17:**
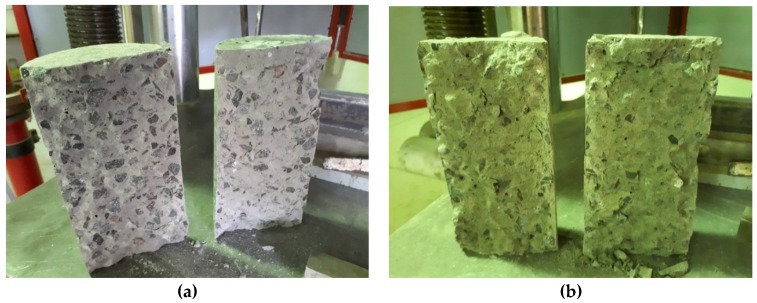
(**a**,**b**) Failure pattern for samples subjected to tension testing.

**Figure 18:**
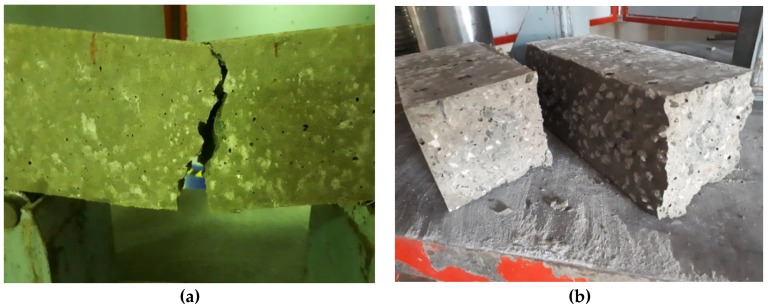
(**a**,**b**) Failure pattern for samples subjected to flexural testing.

Tensile and flexural bending tests failed through the middle as expected for the types of tests being conducted with no tests possessing a failure pattern out of the ordinary. Figure 17 shows the failure pattern of samples subjected to tensile testing, while Figure 18 shows the failure pattern for samples subjected to flexural testing.

## 4. Conclusions

This research may serve as a supporting article for developing GC and HWGC using normal-weight coarse and magnetite aggregates, respectively. The experimental study presented in this research could initially serve for developing and promoting the use of environmentally friendly GC and HWGC by eliminating the carbon emission through OPC in Australia. In addition, the residual strengths of these concretes were examined at high temperatures. All the mixes made with the replacement of magnetite aggregates satisfied the heavyweight concrete classifications and GC classifications. The results presented in this research show the following trends:

For all mixes at 28-day compressive testing, the strength of all samples increases when temperature rises from 100 to 300 °C. Thus, the densification of geopolymer appears to occur at 100 and 300 °C, resulting in an increase in compressive strength by forming larger geopolymer grains.

As the density increases with the replacement of magnetite aggregates, the slump flow diameter decreases. This decrease can be attributed to the difference in the rate of water absorption between normal-weight natural aggregates and magnetite aggregates, where the latter absorbs more water than the normal-weight aggregates. 

A linear trend was observed in slump flow diameter decrements as normal aggregate was replaced by magnetite aggregates.

A significant change in appearance occurred (spalling) when all mix samples were exposed to 900 °C. Severe macro-cracks were observed for samples when the temperature was increased from 600 to 900 °C. This is attributed to the destruction of the cellular structure of geopolymer due to dehydration damage, dimensional instability, and sintering.

Samples at 600 °C exhibited enormous micro cracks on the surface but still retained significant compressive strengths. The sintering process may be considered the main reason for keeping the strength even with microcracking on the surface of samples at 600 °C.

## Figures and Tables

**Table 1:** Properties of fly ash.

Properties	Values
**Chemical**	
CaO	3.30%
SiO_2_	50.40%
Al_2_O_3_	31.50%
Fe_2_O_3_	10.40%
SO_3_	0.10%
MgO	1.10%
Na_2_O	0.30%
K_2_O	0.50%
SrO	<0.1%
TiO_2_	1.90%
P_2_O_5_	0.50%
Mn_2_O_3_	0.20%
Total Alkali	0.60%
**Physical**	
Relative Density	2.29
Moisture	<0.1%
Loss on Ignition	1.10%
Sulfuric Anhydride	0.1%
Chloride Ion	0.00%
Chemical Composition	92.30%
Relative Water Requirement	93.00%
Strength Index	102.0%

**Table 2:** Properties of ground granulated blast furnace slag.

Properties	Values
**Chemical**	
CaO	42%
SiO_2_	31%
S	0.40%
SO_3_	2.40%
MgO	5.70%
Al_2_O_3_	12.70%
FeO	0.80%
MnO	0.10%
Cl	0.01%
Insoluble Residue Content	0.20%
**Physical**	
Specific Gravity	3.0–3.2
Relative Water Requirement	103.00%
Loss on Ignition	0.20%
Relative Strength	100.00%
Temperature Rise	18.8 °C
Fineness (passing 45 µm)	98.00%

**Table 3:** Natural fine sand aggregate distribution.

Sieve Size (µm)	Percentage Passing
1180	100
600	91
300	14.8
150	3.1
75	0

**Table 4:** Properties of natural fine sand (45/50).

Properties	Values
**Chemical**	
SiO_2_	99.86%
Fe_2_O_3_	0.01%
Al_2_O_3_	0.02%
CaO	0.00%
MgO	0.00%
Na_2_O	0.00%
K_2_O	0.00%
TiO_2_	0.03%
MnO	<0.001%
**Physical**	
Loss on ignition	0.01%
Water content (at 105 °C)	<0.001%
American Foundry Society number	47.5

**Table 5:** Ten millimeter naturally crushed aggregate distribution.

Sieve Size	Percentage Passing
13.2 mm	100
9.5 mm	87
6.7 mm	20
4.75 mm	7
2.36 mm	4
1.18 mm	3
600 µm	2
300 µm	0
Moisture Content	0.5
Flakiness Index	24

**Table 6:** **(a)** 10 mm magnetite aggregate distribution; **(b)** properties of 10 mm magnetite aggregate.

**(a)**	
**Sieve Size (mm)**	**Percentage Passing**
16	100
13.2	71.6
9.5	14.7
6.7	0
**(b)**	
**Properties**	**Values**
**Chemical**	
Fe	>95.5%
Si	2.20%
C	0.50%
Mn	2.20%
**Physical**	
Hardness	5.1
Specific Gravity	4.6 g/cm^3^

**Table 7:** Mix proportions.

Mix	Replacement (%)	Binder (kg/m^3^)			Na_2_SiO_3_	NaOH	Water	Aggregate (kg/m^3^)			Admixture SP (l/m^3^)
		Fly Ash	GGBFS	Total Cementitious				Fine Sand	Coarse Aggregate (10 mm)	Magnetite (10 mm)	
M1	0	360	40	400	114.3	45.7	50	650	1210	-	3.25
M2	50	360	40	400	114.3	45.7	50	650	613.33	859.17	3.25
M3	75	360	40	400	114.3	45.7	50	650	307	1288.67	3.25
M4	100	360	40	400	114.3	45.7	50	650	-	1690	3.25

Density of NaOH = 2.13 g/cm^3^. Density of Na_2_SiO_3_ = 1.39 g/cm^3^.

**Table 8:** Fresh property test results.

Mix	Slump Flow Diameter (mm)	Density (kg/m^3^)
M1	608	2364
M2	593	2680
M3	580	2767
M4	575	2861

**Table 9:** Mass loss (percentage) by temperature.

Temperature (°C)	Mass Loss %							
	7 Days				28 Days			
	M1	M2	M3	M4	M1	M2	M3	M4
100	0.38	0.06	0.21	0.11	0.07	0.12	0.24	0.11
300	1.81	0.46	0.52	0.65	0.45	0.73	0.82	0.57
600	5.57	4.65	5.35	4.19	6.46	5.64	5.19	4.12
900	6.16	6.10	6.16	5.98	6.20	6.10	6.20	5.80

**Table 10:** Performance of concrete mixtures at high temperatures.

Mix	Temperature (°C)							
	100		300		600		900	
	7 Day	28 Day	7 Day	28 Day	7 Day	28 Day	7 Day	28 Day
M1	No noticeable spalling occurred						Minimal spalling on top & side surfaces	
M2	No noticeable spalling occurred						Minimal spalling on top & side surfaces	
M3	No noticeable spalling occurred						Minimal spalling on top & side surfaces	
M4	No noticeable spalling occurred						Minimal spalling on top & side surfaces	
